# Structural disadvantage and HIV risk – comparing risk factors between trans women’s partnerships with cis men and trans women sexual partners

**DOI:** 10.1186/s12889-025-23871-1

**Published:** 2025-08-16

**Authors:** Erin C. Wilson, Bow Suprasert, Dillon Trujillo, Sofia Sicro, Christopher J. Hernandez, Caitlin M. Turner, Willi McFarland, Sean Arayasirikul

**Affiliations:** 1 San Francisco Department of Public Health, Trans Research Unit for Equity, Center for Public Health Research, 25 Van Ness Ave Ste.710, San Francisco, CA 94102 USA; 2 David Geffen School of Medicine, University of California Los Angeles, Los Angeles, USA; 3 Department of Epidemiology and Biostatistics, University of California San Francisco, San Francisco, USA; 4 Department of Health, Society, and Behavior, University of California Irvine, Irvine, USA

**Keywords:** Trans women, Sexual partners, HIV risk, Structural disadvantage, Stigma, Social determinants of health

## Abstract

**Introduction:**

Little is known about differences in HIV risk for trans women by partner gender, particularly with respect to social determinants of health and partner-level factors that affect behavior. We examined differences in demographic, social determinants, and HIV-related risk behaviors for trans women with cisgender men and trans women sexual partners.

**Materials and methods:**

Data are from a cross-sectional survey of trans women and their sexual partners conducted between April 2020 and January 2021. Interviews were held remotely via videoconference during shelter-in-place ordinances due to the Covid-19 pandemic. This analysis characterized associations between HIV risk and preventive behaviors comparing trans women with cisgender men partners to trans women with trans women partners.

**Results:**

A total of 336 sexual partners were identified from 156 trans women. Trans women with cisgender men partners were significantly more likely to be from racial/ethnic minority populations and all Black/African American and Latina trans women participants had cisgender men partners only. Trans women with cisgender men partners had significantly less education and employment and more incarceration and recidivism than trans women with trans women partners. Trans women and their cisgender men partners had shared experiences of unstable housing, incarceration, and HIV. Trans women with cisgender men partners reported significantly more sex exchange partners, receptive condomless sex, and HIV compared to trans women with trans women partners.

**Conclusions:**

Trans women with cisgender men sexual partners faced higher HIV risk than trans women with trans women sexual partners. These risks may be related to the social and economic drivers that both trans women and their cisgender men partners faced, namely structural racism that may explain barriers to education and employment, along with incarceration and recidivism. Interventions focused on economic stability, workforce development and post incarceration re-entry housing and employment support for trans women and their cisgender men partners may have the most impact on reducing HIV risk and incidence.

## Introduction

Trans women have a disproportionate burden of HIV throughout the world, yet the proximal cause of HIV acquisition is largely unknown. Although links between large structural factors like anti-trans discrimination and HIV risk are well demonstrated, less is known about the impact of structural drivers on the contexts and behaviors of trans women and their sexual partners [1, 2].

To date, little research has been conducted to examine the risk environment for trans women and their sexual partners. A systematic review of cisgender men (hereafter, cis men) sexual partners of trans women estimated an HIV prevalence of 30.6%, with almost half (46.1%) engaging in condomless anal sex with trans women [3]. A recent online study of cis men who have sex with trans women found that cis men who identify as heterosexual were more likely to report exchange sex, briefer relationships, sex outside partnerships, and less pre-exposure prophylaxis (PrEP) use than cis men with other sexual identities [4]. Our prior study found that African American and Latina trans women report having sexual partners of the same race, which gives some insight into their sexual networks [5]. Other studies have shown that trans women are most likely to engage in sexual risk behavior with cis men partners and primary partners [2, 6, 7]. Research with other populations suggests that condomless sex is more common in primary partnerships with age and income discrepancies [8], and dependent on relationship length and seriousness [9], and intimacy and trust [10]. In addition, trans women may have power imbalances in relationships due to stigma and unmet basic needs, which may result in sexual risk behavior [11]. Yet, few data exist to explain why and how patterns in partnering result in HIV risk.

Social determinants of health are particularly relevant factors for understanding HIV among trans women due to the multiple identity-based stigmas they face [12]. Stigma may impact access to PrEP and HIV care for both trans women and their sexual partners, which impacts risk within sexual networks [13]. HIV research with trans women finds disproportionately high rates of HIV among African American/Black and Latina trans women [14]. The effects of structural racism on mass incarceration, residential segregation, and socio-economic disadvantage may explain individual HIV risk and risk within sexual partnerships, as has been found in other populations [15, 16].

As gender and partnership types continue to expand, so do the dynamics and risks within partnerships among trans women. The present study was conducted to compare structural disadvantage and HIV risk between trans women with cis men partners and trans women with trans women partners. We also examined differences in demographic, behavioral and social determinants of health for trans women by partnership type. We tested the hypothesis that social determinants of health and HIV risk and preventive behaviors were significantly worse for trans women with cis men partners. We also provide data to fill a major gap in research on the social determinants of health for the partners of trans women.

## Methods

Data for this analysis were collected from surveys conducted from April 2020 to January 2021. Surveys were interviewer-administered via Zoom during shelter-in-place ordinances due to the Covid-19 pandemic. Trans women were recruited through word-of-mouth, social media advertisements, and outreach on dating apps. Participants received $50 for participating in the survey, and $20 for referring additional participants. Trans women were eligible for the study if they were 18 years of age or older, identified as a trans woman, had a trans woman and/or cis man sexual partner, lived in California, and spoke English or Spanish.

### Measures

Trans women participants were asked demographics questions such as gender identity, sexual orientation, race and ethnicity, and social determinants of health such as education, employment, age, incarceration, housing stability, income, health insurance, and HIV status of themselves and their sexual partners.

Data on sexual partners reported by trans women relied on their knowledge of up to their 3 most recent partners in the last 12 months. The number of sexual partners was assessed by “How many people did you have vaginal or anal sex with in the past 12 months?”. Participants were also asked “In the past 12 months, what types of sexual partners have you had vaginal or anal sex with?”. Options were (1) main, (2) causal, and (3) exchange. Two additional questions were also asked to assess (1) number of exchange partners and (2) whether trans women exchanged sex for drugs or money in the past 3 months.

HIV risk and preventive behaviors were assessed via self-report. We assessed HIV risk behaviors such as insertive condomless anal sex, receptive condomless anal sex, and insertive and receptive condomless anal sex while using substances in the past 12 months. Concurrent sex was assessed by the following questions; “During the time you were having a sexual relationship with your sexual partners, did you have sex with other people?” (Yes/No) and “As far as you know, during the time you were having a sexual relationship with your partners, did they have sex with other people?”. Options to the second question were (1) Definitely did not, (2) Probably did not, (3) Probably did, and (4) Definitely did. Yes responses to the first question and an answer of “probably did” (3) or “definitely did” (4) to the second question were classified as having concurrent sex. Injection drug use was assessed by asking “Have you injected substances in the past 6 months?”.

We assessed HIV preventive behaviors such as HIV testing, PrEP use, and knowledge of sexual partner’s HIV status. History of HIV testing was asked as “Have you ever been tested for HIV?”. Participants were asked if they were living with HIV or not. PrEP use ever and in the last six months was assessed. For those who responded that they were living with HIV, engagement in HIV care was assessed with questions on initial linkage, current healthcare engagement, and whether they were taking their HIV medications. Viral load was assessed with the question, “Was your most recent viral load detectable or undetectable?”. HIV status of the participants’ partner was asked as “To the best of your knowledge, what is your sexual partner’s HIV status?”. Participants were also asked “How do you know their (i.e., your partner's) HIV status?”. Options were (1) They told me, (2) They showed me their HIV test results, (3) We got tested together, and (4) Other. Only responses 2 and 3 were used for the analysis.

The partnership-level data in this study is egocentric and was therefore based on trans women’s perception and knowledge of their partners. For this analysis, only the three most recent partners were used to create an aggregate of trans women partnership data and to classify trans women into two groups. Gender was asked as “What is your partner’s gender?”. Options were (1) cis man and (2) transgender women. Responses of "don’t know", "refused to answer", or "not applicable" were coded as missing and removed from further analyses. Gender of their three most recent partners were used to classify trans women into trans women with cis men partners. Specifically, if at least one of their three most recent sexual partners was a cis man, they were categorized as a trans woman who has sex with cis men. Trans women were categorized as a trans woman with trans women partners if none of their three most recent partners was a cis man.

### Data analysis

 We tested the hypothesis that trans women with cis men partners were more likely to engage in HIV risk behaviors [6, 7] compared to trans women with trans women partners. Chi-squared or Fisher’s exact tests were conducted to assess differences in characteristics and HIV risk and preventive behaviors between trans women with cis men partners and trans women with trans women partners. Student’s t-test or Wilcoxon Signed-Ranks test were conducted for continuous variables such as number of exchanged partners in the past 12 months. Basic demographics of trans women and their three most recent cis men partners were used to identify concordance in socio-demographic factors within pairs of trans women and cis men. This concordance analysis measures the degree of shared experience or alignment between trans women and their cis men sexual partners. A significant level was set at α < 0.05. All statistical analyses were conducted on STATA version 17 software [17]. All participants provided signed informed consent to participate. The study was approved by the Institutional Review Board (IRB) of the University of California, San Francisco (IRB No. 18-26447).

## Results

Table 1 describes characteristics of trans women and up to three of each trans woman’s sexual partners. A total of 336 sexual partners were aggregated from the responses of 156 trans women. Most partners were identified by trans women as being assigned male sex at birth (97.6), cis men (81.9%), straight (59.1%), employed (65.5%), between the ages of 18–39 years old (69.9%), never incarcerated (68.5%), stably housed (75.3%), and HIV negative (89.7%). Sexual partners were identified as mostly white race/ethnicity (42.0%), followed by African American (23.7%) and Latino/a/x (21.3%). Most trans women identified as straight (47.1%), employed (48.1%), between ages 18–39 years old (66.0%), ever incarcerated (53.3%), stably housed (66.2%), and HIV negative (79.5%). Trans women were mostly white (27.6%), multi-racial/ethnic (25%), and Latina/o/x (23.7%).

**Table 1:** Demographic characteristics, social determinants of health, and HIV status of trans women participants (*N* = 156) and their sexual partners (*N* = 336)

	Trans women *N* = 156 (col %)	Sexual partners *N* = 336 (col %)
Sex at birth		
Male	156 (100)	321 (97.6)
Female	0 (0)	8 (2.4)
Gender identity		
Man	0 (0)	262 (81.9)
Transgender woman	115 (73.7)	58 (18.1)
Woman	36 (23.1)	0 (0)
Non-binary	4 (2.6)	0 (0)
Sexual orientation		
Straight/heterosexual	73 (47.1)	185 (59.1)
Gay/lesbian	7 (4.5)	18 (5.8)
Bisexual	19 (12.3)	50 (16.0)
Pansexual	26 (16.8)	34 (10.9)
Queer	22 (14.2)	21 (6.7)
Questioning	2 (1.3)	3 (1.0)
Other	6 (3.9)	2 (0.6)
Race/ethnicity		
African American/Black	22 (14.1)	79 (23.7)
Asian/Pacific Islander	10 (6.4)	14 (4.2)
Latino/a/x	37 (23.7)	71 (21.3)
Native American	3 (1.9)	2 (0.6)
White	43 (27.6)	140 (42.0)
Multiracial/ethnic	39 (25.0)	23 (6.9)
Other	2 (1.3)	4 (1.2)
Employment		
Unemployed	50 (32.1)	40 (15.5)
Employed	75 (48.1)	169 (65.5)
Living on entitlements	10 (6.4)	11 (4.3)
Other	21 (13.5)	38 (14.7)
Age group		
18–29	54 (34.6)	111 (34.1)
30–39	49 (31.4)	117 (35.9)
40–49	21 (13.5)	55 (16.9)
50+	32 (20.5)	43 (13.2)
Had history of incarceration		
No	72 (46.8)	174 (68.5)
Yes	82 (53.3)	80 (31.5)
Housing stability		
Stable (rent/own)	102 (66.2)	226 (75.3)
Unstable (SRO, supportive housing, homeless, shelter, couch surfing or other)	52 (33.8)	74 (24.7)
HIV status		
Negative	124 (79.5)	262 (89.7)
Positive	31 (19.9)	19 (6.5)
Unknown	1 (0.6)	11 (3.8)

Answer don’t know, refuse, not applicable were coded as missing

Table 2 presents data comparing characteristics of trans women in partnerships with trans women to trans women with cis men sexual partners. Of 156 trans women, 130 (85.5%) were classified as having at least one cis man sexual partner, while 22 (14.5%) had trans women partners. Of 130 trans women with cis men partners, more than half identified partners as main (68.5%) and casual (63.0%). One-fourth of trans women with cis men partners identified their partners as exchange partners (25.0%), while only one trans woman with trans women partners had an exchange partner (5.6%). Among 130 trans women with cis men partners, slightly more than half identified as straight/heterosexual (55.0%). No trans women with trans women partners identified as straight/heterosexual and many identified as queer (31.8%), pansexual (27.3%), and gay or lesbian (18.2%). Out of 130 trans women with cis men partners, many identified as Latina/o/x (27.7%) followed by multiracial/ethnic (24.6%), White (21.5%), and African American/Black (16.9%). Most trans women with trans women partners identified as White (63.6%). While few trans women in our study had vaginoplasty (*N* = 18, 11.8%), most with vaginoplasty had cis men sexual partners (*N* = 14, 77.8%).

**Table 2:** Differences between trans women with trans women partners and with Cis gender men partners (*N* = 152)

Characteristics	Trans women with trans women partnerships *N* = 22 (14.5%)	Trans women with cis men partnerships *N* = 130 (85.5%)	*P*-value^a^
Age			
Mean (SD)	33.6 (7.7)	37.5 (12.2)	0.149
Median (IQR)	32 (28, 36)	34.5 (28, 47)	0.323
Age group			0.069
18–29	7 (31.8)	47 (36.2)	
30–39	11 (50)	35 (26.9)	
40–49	3 (13.6)	17 (13.1)	
50+	1 (4.6)	31 (23.9)	
Gender identity			0.124
Transgender woman	8 (36.4)	26 (20.2)	
Woman	13 (59.1)	100 (77.5)	
Non-binary	1 (4.6)	3 (2.3)	
Had vaginoplasty			0.299
No	18 (81.8)	116 (89.2)	
Yes	4 (18.2)	14 (10.8)	
Number of sexual partners, past 12 months			
Mean (SD)	3.7 (3.7)	15.4 (53.1)	0.341
Median (IQR)	2 (1, 5)	3 (2, 5)	0.193
Partner types, past 12 months^b^			
Main	15 (83.3)	63 (68.5)	0.264
Casual	11 (61.1)	58 (63.0)	0.877
Exchange	1 (5.6)	23 (25.0)	0.115
Sexual orientation			**< 0.001**
Straight/heterosexual	0 (0)	71 (55.0)	
Gay/lesbian	4 (18.2)	3 (2.3)	
Bisexual	1 (4.6)	17 (13.2)	
Pansexual	6 (27.3)	20 (15.5)	
Queer	7 (31.8)	14 (10.9)	
Questioning	1 (4.6)	1 (0.8)	
Other	3 (13.6)	3 (2.3)	
Race/ethnicity			**< 0.001**
African American/Black	0 (0)	22 (16.9)	
Asian/Pacific Islander	2 (9.1)	8 (6.2)	
Latino/a/x	0 (0)	36 (27.7)	
Native American	0 (0)	3 (2.3)	
White	14 (63.6)	28 (21.5)	
Multiracial/ethnic	6 (27.3)	32 (24.6)	
Other	0 (0)	1 (0.8)	
Education			**0.036**
Less than undergraduate degree	11 (50.0)	94 (72.3)	
Undergraduate degree or more	11 (50.0)	36 (27.7)	
Employment			**0.018**
Employed	16 (72.7)	59 (45.4)	
Not employed	6 (27.3)	71 (54.6)	
Had history of incarceration			**< 0.001**
No	18 (81.8)	54 (42.2)	
Yes	4 (18.2)	74 (57.8)	
Number of times incarcerated			
Mean (SD)	0.18 (0.4)	4.11 (7.5)	**0.015**
Median (IQR)	0 (0, 0)	1 (0, 6)	**< 0.001**
Housing Stability			0.142
Stable	18 (81.8)	83 (63.9)	
Unstable	4 (18.2)	47 (36.2)	

^a^Chi-squared, Fisher’s exact, T-test, or Wilcoxon Signed-Ranks Test

^b^Multi-responses were allowed

Boldface *p*-values indicate this factor was significant at the level of α < 0.05

Significantly more trans women with trans women sexual partners had a college degree compared to trans women with cis men partners (50.0% vs. 27.7%, *p* = 0.036). Nearly half of trans women with cis men partners reported being employed (45.4%), while most trans women with trans women partners reported being employed (72.7%, *p* = 0.018). Among 59 employed trans women with cis men partners, 5 (8.5%) reported having at least one unemployed partner. Of 16 employed trans women with trans women partners, 1 (6.3%) reported having at least one employed partner. Among 71 unemployed trans women with cis men partners, 43 (60.6%) reported having at least one employed partner. Four of the 6 (66.7%) of unemployed trans women with trans women partners reported having at least one employed partner.

More than half of trans women with cis men partners reported a history of incarceration (57.8%) compared to 18.2% of trans women with trans women partners (*p* < 0.001). On average, trans women with cis men partners were incarcerated 3.93 more times than trans women with trans women partners (mean = 4.11, SD = 7.5 vs. mean = 0.18, SD = 0.4, *p* = 0.015). Although the difference was not statistically significant, more trans women with cis men partners reported unstable housing than trans women with trans women partners (36.2% vs. 18.2%). Among 83 trans women with cis men partners with stable housing, 10 (12.0%) reported having at least one partner who was not stably housed. Of 18 trans women with trans women partners with stable housing, 1 (5.6%) reported having at least one partner who was not stably housed. Among 47 trans women with cis men partners who reported unstable housing, 8 (17.0%) reported having at least one partner who had stable housing. All trans women with trans women partners who were unstably housed reported having at least one partner who had stable housing.

Table 3 shows concordance among the 130 trans women with cis men partners on self-reported race/ethnicity, occupation, age, incarceration, and housing. Most African American/Black (72.7%) and White (64.3%) trans women reported having at least one cis man partner of the same race/ethnicity. No trans women who identified as Asian/Pacific Islander or Native American reported having a cis man partner of the same race/ethnicity. Slightly more than half of trans women in the age range of 40–49 years old reported having cis men partners from different age ranges (52.9%). Slightly more than half of trans women with a history of incarceration had cis men partners who also had a history of incarceration (55.4%). Almost half of trans women who currently lived in a single room occupancy (SRO) hotel had a sexual partner with the same living situation (43%), and of trans women who were currently homeless, 22% had a partner who was also currently homeless. Of 101 trans women with cis men partners who reported that they were not living with HIV, 11 had at least one partner who was living with HIV or their HIV status was unknown (10.9%). Of 28 trans women with cis men partners who reported that they were living with HIV, 57.1% had at least one partner who was not living with HIV or their partner’s status was unknown.

**Table 3:** Concordance in demographic characteristics and social determinants of health among trans women with Cis men sexual partners (*N* = 130)

	Concordance *N*, %
Race/ethnicity	
African American/Black	16 (72.7)
Asian/Pacific Islander	0 (0)
Latino/a/x	21 (58.3)
Native American	0 (0)
White	18 (64.3)
Multiracial/ethnic	11 (34.4)
Other	0 (0)
Age	
Mean (SD)	36.8 (12.6)
Median	32
Age group	
18–29	33 (70.2)
30–39	25 (71.4)
40–49	8 (47.1)
50+	20 (64.5)
History of incarceration	
No	46 (85.2)
Yes	41 (55.4)
Current living situation	
Rent/own	72 (86.8)
SRO	7 (43.8)
Supportive housing	2 (16.7)
Homeless/shelter/couch	2 (22.2)
Other	2 (20)
Housing Stability	
Stable	72 (86.8)
Unstable	13 (15.3)
HIV Status	
Negative	90 (89.1)
Positive	12 (42.9)
Unknown	1 (100)

 Table 4 shows comparison in HIV risk and preventive behaviors between trans women in partnerships with trans women partners to trans women with cis men sexual partners. Trans women with cis men partners were significantly more likely to report exchange sex for drugs or money in the past 3 months (27.7 vs. 4.6%, *p* = 0.016) and receptive condomless anal sex in the past 3 months (67.7% vs. 31.8%, *p* = 0.001) than trans women with trans women partners. Eight trans women injected any drugs in the past 6 months, and all were trans women with cis men partners. All trans women living with HIV were trans women with cis men partners. Of all trans women with cis men partners who were living with HIV, most (92.9%) received HIV care and (85.7%) were virally suppressed.

**Table 4:** HIV-related risk and preventive factors by partnership type comparing trans women in sexual partnerships with Cis men partnerships to trans women with trans women partnerships (*N* = 152)

	Trans women with trans women partnerships *N* = 22	Trans women with cis men partnerships *N* = 130	*P*-value
HIV risk behaviors and HIV status			
Exchanged sex for drugs or money, past 3 months^a^	1 (4.6)	36 (27.7)	**0.016**
Insertive condomless anal sex, past 12 months^a^	4 (18.2)	24 (18.5)	1.000
Receptive condomless anal sex, past 12 months^a^	7 (31.8)	88 (67.7)	**0.001**
Insertive or receptive condomless anal sex while using substances, past 12 months^a^	5 (22.7)	56 (43.1)	0.072
Had concurrent sex	21 (95.5)	99 (83.2)	0.197
Injected drugs, past 6 months^a^	0 (0)	8 (6.2)	0.603
Self-reported HIV positive	0 (0)	28 (21.5)	**0.014 **
With the most recent partner who is HIV positive^b^	0 (0)	12 (10.7)	0.215
HIV preventive behaviors			
Ever engaged in HIV care	--	28 (21.5)	--
Currently receive HIV care	--	26 (20.0)	--
Currently on ART	--	26 (20.0)	--
Ever on PrEP^a^	10 (45.5)	67 (51.5)	0.598
On PrEP, past 6 months^a^	5 (22.7)	44 (33.9)	0.076
Knew HIV status of the most recent partner	6 (28.6)	37 (33.0)	0.688
Ever been tested for HIV	22 (100)	129 (99.2)	1.000
Virally suppressed^a^	--	24 (98.4)	--

^a^Chi-squared, Fisher’s exact, T-test, or Wilcoxon Signed-Ranks Test

Boldface *p*-values indicate this factor was significant at the level of α < 0.05

## Discussion

HIV risk and structural disadvantage was highest among trans women with cis men partners. We found that African American/Black, Latina and Native American trans women in our study had only cis men partners. Structural barriers likely rooted in racism were significantly more prevalent among trans women with cis men partners compared to those with trans women partners.

We found that significantly more trans women with cis partners engaged in sex work, pointing to income insecurity [18]. Most trans women engage in sex work due to gender-based discrimination leaving work in the information sector as the only way to meet survival needs [19, 20]. Some also do sex work to meet gender-related surgery goals and for better pay than in other jobs [19]. Although sex work can be a source of income [21], it comes with threats of violence, criminalization, mental distress, and risk for HIV infection [19, 20, 22]. Trans women who do sex work may be at risk of HIV for a variety of reasons, including the number of partners they have increasing chances of HIV exposure, inability to negotiate condom use, and sexual violence [23].

We also found that although more than half of trans women with cis men partners had stable housing, one third of them did not. Of trans women who lived in single room occupancy hotels (SROs), 43% of their cis men partners had the same living situation. Living in an SRO has been associated with numerous health risks including substance use, mental illness, and HIV risk [24, 25]. For trans women, unstable housing is associated with poor HIV care outcomes [26, 27] and elevated risk behavior [28], along with incarceration and low income [29]. Half of trans women with cis men partners who had a history of incarceration also had partners with a history of incarceration. Incarceration is associated with under-employment, as we observed in our study, which ultimately effects income and lifetime earnings leading to poverty for many people [30]. A study conducted in Los Angeles with heterosexually identified cis men who occasionally had sex with trans women found similar levels of structural disadvantage as most had low income, high unstable housing, low education, and more than 80% had been incarcerated [31]. Data published from a national dataset of trans women similarly found disparities in higher structural syndemic conditions among trans women who are African American/Black and Hispanic along with higher risk for HIV [32].

Much of the structural disadvantage we observed among trans women with cis men partners may be explained by the differences in race/ethnicity by partnership type. Trans women with cis men sexual partners were significantly more likely to be from minoritized racial/ethnic accepted groups who faced higher rates of structural racism observed in lower educational attainment, lower employment and more incarceration compared to mostly white and mixed-race trans women with trans women partners. It has long been recognized that HIV is a pandemic of social disadvantage [33]. A large body of research demonstrates how structural systems affect HIV risk and access to and engagement in HIV prevention and care among populations most impacted by HIV [16]. These results situate racial/ethnic minoritized trans women’s risk within their partnering with similarly racial/ethnic minoritized cis men who may face the same structural disadvantage, resulting in amplified risk for both partners.

We also found that trans women in partnerships with cis men were significantly more likely to report condomless receptive anal sex than trans women with trans women partners. An important contextual factor from this analysis is that almost all partners of trans women in our dataset were assigned male sex at birth, yet condomless receptive anal sex was more common among trans women with cis men sexual partners. Research has established that the primary mode of HIV risk for trans women is condomless receptive anal sex [5, 34, 35]. Trans women with cis men partners were also significantly more likely to identify as heterosexual than trans women with trans women sexual partners, and sexual risk may be particularly prevalent in trans women’s sexual relationships with cis heterosexual men partners. In a study of 80 trans women who have lived with HIV for anywhere from 4 to 34 years, they reported identifying as trans women when they acquired HIV and most acquired it from their heterosexual cis men partners [36]. An online study of cis men with trans women sexual partners found that heterosexual cis men engaged in significantly more sexual risk behaviors compared to gay, bisexual, and queer cis men partners [4]. Research with cis men living with HIV found they were less likely to use condoms with trans women than cis women or men sexual partners because they assumed trans women were already living with HIV. Policy and intervention approaches are needed that recognize that trans women most at risk of HIV are likely to identify as heterosexual, have cis men sexual partners, and face significant structural disadvantage.

Notably, there were no significant differences in recent HIV testing or PrEP among trans women with cis men partners compared to those with trans women partners. Indications of equal access to HIV prevention services for all partner types is positive and speaks to effectiveness in focused efforts in California to meet trans women’s HIV prevention needs. We also identified high sexual mixing by race, which may serve as a preventive factor for trans women of color in partnerships with cis men as has been found among Black MSM [37].

The primary limitation to this study was recruitment challenges we encountered from having to move the study from in-person to remote due to Covid-19 shelter in place ordinances. This change limited our ability to obtain HIV biomarkers via rapid tests as was initially proposed and to rely on self-reported data. The small sample size is also relatively small, as is common in research with trans populations who represent about 0.6% of the overall U.S. population [38]. Due to small numbers in the groups, we recommend caution in interpreting the results of the group comparisons. We note that datasets like this are rare, and although there are limitations due to the small sample size, these data can serve as a building block to future research like network studies that are needed to fully understand the direct HIV risks trans women face and to determine how to better engage their partners in research and programs. Despite these challenges, findings from this study fill important gaps in understanding trans women’s HIV risks within partnerships with cis men and the structural disadvantage cis men partners of trans women face.

## Conclusions

Research to improve impact in HIV prevention for trans women will also need to incorporate interventions for their cis men sexual partners. [39, 40] And without identifying and addressing structural barriers related to racism, including within partnerships, the U.S. will not be able to end the HIV epidemic [41].

## Data Availability

The data that support the findings of this study are available on request from the corresponding author. The data are not publicly available due to privacy or ethical restrictions.

## References

[1] Bradford J, Reisner SL, Honnold JA, Xavier J. Experiences of transgender-related discrimination and implications for health: results from the Virginia transgender health initiative study. Am J Public Health. 2013;103(10):1820–9. 23153142 10.2105/AJPH.2012.300796 PMC3780721

[2] Poteat T, Cooney E, Malik M, Restar A, Dangerfield DT 2nd, White J. HIV Prevention Among Cisgender Men Who have Sex with Transgender Women. AIDS Behav. 2021;25(8):2325–35. 10.1007/s10461-021-03194-z. Epub 25 Feb 2021. PMID: 33634354; PMCID: PMC8222096. 10.1007/s10461-021-03194-z PMC8222096 33634354

[3] Restar AJ, Operario D. The missing trans women of science, medicine, and global health. Lancet. 2019;393(10171):506–8. 30739673 10.1016/S0140-6736(18)32423-1

[4] Skeen SJ, Starks TJ, Jimenez RH, Rendina HJ, Cain D. Heterosexual cisgender men partnered with transgender women exhibit higher HIV/STI sexual risk than their gay, bisexual, and queer counterparts: findings from a U.S.-based convenience sample recruited online. AIDS Behav. 2021;25(10):3279–91. 34050403 10.1007/s10461-021-03314-9 PMC10062375

[5] Wilson EC, Santos GM, Raymond HF. Sexual mixing and the risk environment of sexually active transgender women: data from a respondent-driven sampling study of HIV risk among transwomen in San Francisco, 2010. BMC Infect Dis. 2014;14:430. 25100405 10.1186/1471-2334-14-430 PMC4132923

[6] Bockting WO, Robinson BE, Rosser BR. Transgender HIV prevention: a qualitative needs assessment. AIDS Care. 1998;10(4):505–25. 9828969 10.1080/09540129850124028

[7] Nemoto T, Operario D, Keatley J, Villegas D. Social context of HIV risk behaviours among male-to-female transgenders of colour. AIDS Care. 2004;16(6):724–35. 15370060 10.1080/09540120413331269567

[8] Mustanski B, Lyons T, Garcia SC. Internet use and sexual health of young men who have sex with men: a mixed-methods study. Arch Sex Behav. 2011;40(2):289–300. 20182787 10.1007/s10508-009-9596-1 PMC2914836

[9] Mustanski B, Newcomb ME, Clerkin EM. Relationship characteristics and sexual risk-taking in young men who have sex with men. Health Psychol. 2011;30(5):597–605. 21604883 10.1037/a0023858 PMC3184611

[10] Appleby PR, Miller LC, Rothspam S. The paradox of trust for male couples: when risk taking is a part of loving. Pers Relat. 1999;6:81–93.

[11] Poteat T, Ackerman B, Diouf D, Ceesay N, Mothopeng T, Odette KZ, et al. HIV prevalence and behavioral and psychosocial factors among transgender women and cisgender men who have sex with men in 8 African countries: a cross-sectional analysis. PLoS Med. 2017;14(11):e1002422. 29112689 10.1371/journal.pmed.1002422 PMC5675306

[12] Poteat T, Malik M, Scheim A, Elliott A. HIV prevention among transgender populations: knowledge gaps and evidence for action. Curr HIV AIDS Rep. 2017;14(4):141–52. 28752285 10.1007/s11904-017-0360-1 PMC5896563

[13] Wilson EC, Hernandez CJ, Scheer S, Trujillo D, Arayasirikul S, Sicro S, et al. Improved PrEP awareness and use among trans women in San Francisco, California. AIDS Behav. 2022;26(2):596–603. 34390435 10.1007/s10461-021-03417-3 PMC8813678

[14] Lee K, Trujillo L, Olansky E, Robbins T, Brune CA, Morris E, et al. Factors associated with use of HIV prevention and health care among transgender women - seven urban areas, 2019–2020. MMWR Morbidity Mortal Wkly Rep. 2022;71(20):673–9. 10.15585/mmwr.mm7120a1 PMC9129907 35588092

[15] Bohl DD, Raymond HF, Arnold M, McFarland W. Concurrent sexual partnerships and racial disparities in HIV infection among men who have sex with men. Sex Transm Infect. 2009;85(5):367–9. 19773457 10.1136/sti.2009.036723

[16] Bowleg L, Malekzadeh AN, Mbaba M, Boone CA. Ending the HIV epidemic for all, not just some: structural racism as a fundamental but overlooked social-structural determinant of the US HIV epidemic. Curr Opin HIV AIDS. 2022;17(2):40–5. 35102051 10.1097/COH.0000000000000724 PMC9109814

[17] StataCorp. Stata statistical software. Release 17. College Station: StataCorp LLC; 2021.

[18] Sausa LA, Keatley J, Operario D. Perceived risks and benefits of sex work among transgender women of color in San Francisco. Arch Sex Behav. 2007;36(6):768–77. 17674180 10.1007/s10508-007-9210-3

[19] Fisher MR, Turner C, McFarland W, Breslow AS, Wilson EC, Arayasirikul S. Through a Different Lens: Occupational Health of Sex-Working Young Trans Women. Transgend Health. 2023;8(2):200–6. 10.1089/trgh.2021.0109. PMID: 37013087; PMCID: PMC10066761. 10.1089/trgh.2021.0109 PMC10066761 37013087

[20] Wirtz AL, Poteat TC, Malik M, Glass N. Gender-Based Violence Against Transgender People in the United States: A Call for Research and Programming. Trauma Violence Abuse. 2020;21(2):227–41. 10.1177/1524838018757749. Epub 13 Feb 2018. PMID: 29439615. 10.1177/1524838018757749 29439615

[21] Logie CH, Abramovich A, Schott N, Levermore K, Jones N. Navigating stigma, survival, and sex in contexts of social inequity among young transgender women and sexually diverse men in Kingston, Jamaica. Reprod Health Matters. 2018;26(54):72–83. 30475167 10.1080/09688080.2018.1538760

[22] Aggarwal NK, Consavage KE, Dhanuka I, Clement KW, Bouey JH. Health and health care access barriers among transgender women engaged in sex work: a synthesis of U.S.-based studies published 2005–2019. LGBT Health. 2021;8(1):11–25. 33297834 10.1089/lgbt.2019.0243

[23] Shannon K, Crago AL, Baral SD, Bekker LG, Kerrigan D, Decker MR, et al. The global response and unmet actions for HIV and sex workers. Lancet. 2018;392(10148):698–710. 30037733 10.1016/S0140-6736(18)31439-9 PMC6384122

[24] Evans L, Strathdee SA. A roof is not enough: unstable housing, vulnerability to HIV infection and the plight of the SRO. Int J Drug Policy. 2006;2:115–7.

[25] Knight KR, Lopez AM, Comfort M, Shumway M, Cohen J, Riley ED. Single room occupancy (SRO) hotels as mental health risk environments among impoverished women: the intersection of policy, drug use, trauma, and urban space. Int J Drug Policy. 2014;25(3):556–61. 24411945 10.1016/j.drugpo.2013.10.011 PMC4014526

[26] Baguso GN, Turner CM, Santos GM, Raymond HF, Dawson-Rose C, Lin J, et al. Successes and final challenges along the HIV care continuum with transwomen in San Francisco. J Int AIDS Soc. 2019;22(4):e25270. 31037858 10.1002/jia2.25270 PMC6488760

[27] Chiu I, Leathers M, Cano D, Turner CM, Trujillo D, Sicro S, Arayasirikul S, Taylor KD, Wilson EC, McFarland W. HIV prevalence, engagement in care, and risk behavior among trans women, San Francisco: Evidence of recent successes and remaining challenges. Int J STD AIDS. 2022;33(12):1029–37. 10.1177/09564624221111278. Epub 11 Jul 2022. PMID: 35816424; PMCID: PMC9607899. 10.1177/09564624221111278 PMC9607899 35816424

[28] Cano D, Leathers M, Chiu I, Turner C, Trujillo D, Sicro S, et al. The effects of HIV-related structural discrimination on mental health outcomes for trans women. In: 24th International AIDS Conference. Montreal: 2022.

[29] Baguso GN, Santiago-Rodriguez E, Gyamerah AO, Wilson EC, Chung C, McFarland W, et al. Mental distress and use of stimulants: analysis of a longitudinal cohort of transgender women. LGBT Health. 2023;10(3):228–36. 10.1089/lgbt.2021.0192 PMC10079245 36301245

[30] Craigie T-A, Grawert A, Kimble C. Conviction, imprisonment, and lost earnings: how involvement with the criminal justice system deepens inequality. New York University School of Law; 2020. https://www.brennancenter.org/sites/default/files/2020-09/EconomicImpactReport_pdf.pdf.

[31] Reback CJ, Kaplan RL, Bettcher TM, Larkins S. The role of the illusion in the construction of erotic desire: narratives from heterosexual men who have occasional sex with transgender women. Cult Health Sex. 2016;18(8):951–63. 26967172 10.1080/13691058.2016.1150515 PMC4914457

[32] Hershow RB, Trujillo L, Olansky E, Lee K, Agnew-Brune C, Wejnert C, et al. Structural and psychosocial syndemic conditions and condomless anal intercourse among transgender women - national HIV behavioral surveillance among transgender women, seven urban areas, United States, 2019–2020. MMWR Suppl. 2024;73(1):21–33. 38261572 10.15585/mmwr.su7301a3 PMC10826687

[33] Pellowski JA, Kalichman SC, Matthews KA, Adler N. A pandemic of the poor: social disadvantage and the U.S. HIV epidemic. Am Psychol. 2013;68(4):197–209. 23688088 10.1037/a0032694 PMC3700367

[34] Nemoto T, Bodeker B, Iwamoto M, Sakata M. Practices of receptive and insertive anal sex among transgender women in relation to partner types, sociocultural factors, and background variables. AIDS Care. 2014;26(4):434–40. 24160715 10.1080/09540121.2013.841832

[35] Bowers JR, Branson CM, Fletcher J, Reback CJ. Differences in substance use and sexual partnering between men who have sex with men, men who have sex with men and women and transgender women. Cult Health Sex. 2011;13(6):629–42. 21442499 10.1080/13691058.2011.564301

[36] Wilson EC, Hernandez CJ, Arayasirikul S, Scheer S, Trujillo D, Sicro S, et al. Their own words: how trans women acquired HIV infection. AIDS Behav. 2022;26(6):2091–8. 35031891 10.1007/s10461-021-03555-8

[37] Millett GA, Peterson JL, Wolitski RJ, Stall R. Greater risk for HIV infection of black men who have sex with men: a critical literature review. Am J Public Health. 2006;96(6):1007–19. 16670223 10.2105/AJPH.2005.066720 PMC1470628

[38] Herman JL, Flores AR, O’Neill KK. How many adults and youth identify as transgender in the united states? Williams Institute. UCLA School of Law; 2022. https://williamsinstitute.law.ucla.edu/wp-content/uploads/Trans-Pop-Update-Jun-2022.pdf.

[39] Fletcher JB, Reback CJ. Associations between gender identity control, gender identity non-verification, and health risks among trans women of color living with HIV. Arch Sex Behav. 2022;51(4):2003–14. 35445282 10.1007/s10508-021-02264-6 PMC10462393

[40] Scott D. Stress and coping amongst cisgender male partners of transgender women. Cult Health Sex. 2022;24(2):196–209. 33236670 10.1080/13691058.2020.1825814

[41] Bowleg L. The problem with the phrase women and minorities: intersectionality—an important theoretical framework for public health. Am J Public Health. 2012;102(7):1267–73. 22594719 10.2105/AJPH.2012.300750 PMC3477987

